# The effect of a Schema-based method on correcting persistent errors in mental arithmetic: an experimental study

**DOI:** 10.3389/fpsyg.2024.1276914

**Published:** 2024-05-20

**Authors:** Shufang Chen, Dawei Liu, Huifen Yan, Yong Ma

**Affiliations:** ^1^College of Mathematics and Statistics, Hubei University of Education, Wuhan, China; ^2^College of Teacher Education, Hubei University of Education, Wuhan, China; ^3^Wuhan Luoyi Education Research Institute, Wuhan, China

**Keywords:** elementary school students, mental arithmetic, memory retrieval, schema theory, simple addition

## Abstract

**Introduction:**

Arithmetic calculation is a fundamental skill for mathematical learning and daily life. However, elementary school students often make errors in practice.

**Methods:**

Grounded in the schema theory and the memory retrieval theory of mental arithmetic, this study employs a controlled experiment to investigate the effect of a schema-based method in correcting persistent errors in mental arithmetic, specifically in the context of simple addition operations. The experimental group utilizes a schema-based method to help participants rectify incorrect answers in memory retrieval, while the control group did not receive this treatment.

**Results:**

The results showed that significant differences emerged between the experimental and control groups in both the post-test performance and the reduction of persistent error count, indicating that the experimental group had rectified incorrect answers in memory; and persistent errors in simple addition were indeed caused by interference from incorrect answers during memory retrieval; and the schema-based method proves to be effective.

**Discussion:**

The findings of this study contribute to enhancing practical mental arithmetic instruction, assisting students in correcting relevant errors, and improving their mental arithmetic abilities. Not only does it offer directive guidance for teaching practices, but it also provides an enlightening reference for promoting innovative teaching methods.

## 1 Introduction

The arithmetic calculation, as a fundamental part of mathematics learning, serves as its foundation and helps students develop logical reasoning abilities. Meanwhile, it is also an essential life skill. Overly slow calculating speeds or frequently occurring arithmetic errors will inevitably lead to difficulties in their mathematics learning and present challenges for teachers in their instruction. National Council of Teachers of Mathematics, Commission on Standards for School Mathematics (2006) explicitly highlights the importance of early-acquired arithmetic ability in the elementary school curriculum and teaching. Therefore, improving the students’ speed and accuracy in arithmetic calculations is of paramount importance for both mathematics education and the individuals’ development.

Unlike general arithmetic calculations, mental arithmetic is a crucial cognitive activity in daily life, emphasizing the process of performing arithmetic operations without the use of external tools such as pens, paper, or calculators (Maclellan, 2001; Erdem, 2017). Grounded in cognitive structures of numerical understanding, mental arithmetic enhances comprehension of numerical systems and operations (Lemonidis, 2015; Jurić and Pjanić, 2023). Prior to the 1970s, cognitive psychology did not consider mental arithmetic as a research topic. Notwithstanding, some earlier works had explored the relationship between mathematics (arithmetic) and psychology. For example, the German philosopher and mathematician Husserl (1891) investigated the psychological foundation of mathematical thinking and how these thoughts were reflected in the logical structure of mathematics. Thorndike (1922) examined the process of human learning and understanding of arithmetic, as well as the application of psychology in education. However, it was not until the publication of the paper, *A chronometric analysis of simple addition* by Groen and Parkman (1972) that cognitive psychology officially recognized the beginning of its research on mental arithmetic, leading to gradually increasing interest in pertinent studies thereafter. Nowadays, researchers have recognized the importance of mental arithmetic and have successively made attempts to explore the impact of mental arithmetic abilities on elementary school students (Varol and Farran, 2007).

At the same time, considerable research findings about arithmetic errors have been revealed, such as the phenomenon of Post-Error Slowing, where individuals exhibit a slowing down after making errors in mental arithmetic. The slowing down reflects both internal reactions to errors and correction processes, with higher-accuracy individuals who focus more on internal reactions showing a more pronounced slowing effect (Steinborn et al., 2012). Recently, Sokolov (2023) has explored the reasons for arithmetic errors occurring during children’s developing stage of mathematical concepts. Lestiana et al. (2017) delved into students’ errors in fraction calculations, and classified the errors into three types based on Brown and Skow’s (2016) framework: factual errors, procedural errors, and computational errors. In this context, the present study aims to investigate the effect of a schema-based method in rectifying persistent errors in mental arithmetic through a controlled experiment. The method is designed to address the issue of persistent errors, and will be illuminated herein, to provide a more comprehensive understanding of errors to research on mental arithmetic.

## 2 Literature review

### 2.1 Mathematical arithmetic errors

For the study on arithmetic errors, it is a crucial step to identify the types of errors (Reisman, 1972; Ashlock, 1986; Morales, 2014; Pankow et al., 2018). The systematic handling of errors made by students is a vital stage in teacher’s work, involving the spotting and explanation of errors (Rozak and Nurwiani, 2020; Ngcobo, 2021; Sanina and Sokolov, 2021). In further research on arithmetic errors, scholars have summarized the error types, including Errors of perseveration, Errors of association, Errors of interference, knowledge errors, and comprehension errors, etc. (Radatz, 1979; Sarwadi and Shahrill, 2014). The classic classification of error mentioned in *Human Error* includes mistakes, false-rule applications, and slips of action (Reason, 1990).

Moreover, in mental arithmetic, simple addition serves as the foundation of arithmetic proficiency and the cornerstone for developing mathematical skills (Ashcraft, 1992; National Council of Teachers of Mathematics, Commission on Standards for School Mathematics, 2000; Peng et al., 2017). Obtaining the actual mastery of basic addition facts is one of the children’s learning objectives (Office for Economic Co-operation and Development, 2014). The development of complex addition and multiplication skills relies on proficiency in simple addition (Geary and Brown, 1991; Geary et al., 2004). Thus, investigating errors in simple addition is essential for developing students’ mental arithmetic abilities.

This paper aims to explore effective methods for addressing the problem of persistent errors in simple addition. As for persistent errors, Myers (1924) first introduced the concept. He made regular tests of basic arithmetic for students and found that they made the same errors repeatedly. For example, students might often write the result of 7 + 4 as 10 instead of any other digit. This paper posits that this phenomenon is related to interference from incorrect answers during memory retrieval. Memory retrieval involves extracting information from memory (Jensen and Whang, 1994). When subjects solve arithmetic problems involving single-digit numbers, they retrieve arithmetic answers from long-term memory (Geary and Brown, 1991; FÜrst and Hitch, 2000; Noël et al., 2001; Geary et al., 2004; Swanson et al., 2008; Paul and Reeve, 2016).

### 2.2 The relationship between memory retrieval and persistent errors

The efficiency of mental calculation is associated with two critical factors: the organization of simple arithmetic facts in long-term memory, and the processing of information in working memory (De Rammelaere et al., 1999; Ding et al., 2019). The hypothetical model proposed by Jonides (1995) regarding the mental calculation process indicates that working memory and long-term memory are concurrently involved. Long-term memory provides various mental calculation knowledge and solution strategies, while working memory processes and stores information during mental calculation (Jonides, 1995; DeStefano and LeFevre, 2004). Research suggests that students are apt to develop their own mental arithmetic strategies rather than solely rely on the methods imparted at school. Given the fact that some students may adopt inefficient mental calculation strategies, learning systematic mental arithmetic strategies is supposed to be emphasized (Joung, 2018; Baranyai et al., 2019; Månsson, 2023).

For mental calculation, two classic strategies are commonly employed: counting strategy and memory retrieval strategy. The transition from the counting strategy to the memory retrieval strategy typically occurs in the third grade of primary school when half of the students use counting and the other half use memory retrieval, approximately. After the third grade, the role of memory retrieval will turn to be increasingly pronounced (Ashcraft and Fierman, 1982; Geary and Brown, 1991; Lucangeli et al., 2003). Existing memory retrieval models can be categorized into two types: table-search models and associate models. Table-search models propose that arithmetic knowledge in long-term memory is organized in the form of tables, where entries represent the sums of numbers (Ashcraft and Battaglia, 1978; Butterworth et al., 2001; Verguts and Fias, 2005). Therefore, the total steps depend on the sum of the numbers, which often leads to a slow and cumbersome process.

Associate models include network retrieval models, distribution of associations models, and network interference models. The network retrieval model posits that answers to simple addition problems are retrieved from a memory network. The addition network is represented as a matrix stored at the intersection points of entry nodes corresponding to the two addends (Ashcraft and Battaglia, 1978; Ashcraft, 1992). In this model, the difficulty in retrieving arithmetic answers depends on the strength of the association between numbers and answers which increases with the frequency of retrieval. The distribution of associations model suggests that the difficulty depends on the interference from other answers, originating from the stage of acquiring arithmetic skills and being related to incorrect answers (Siegler, 1988). The network interference model attempts to combine associative strength and interference to explain adult problem size effects while simulating the developmental changes in mental calculation skills, to elucidate the transition from counting to memory extraction (Zbrodoff, 1995).

Memory retrieval is considered as a strategy that can easily achieve high accuracy and high speed of calculation (Steel and Funnell, 2001; Ophuis-Cox et al., 2023). As evident from the previous discussion, the difficulty in memory retrieval is related to associative strength and interference. Therefore, it can be inferred that the persistent errors made by students are attributed to weak associations between equations and correct answers, coupled with interference from incorrect answers. To facilitate a more accurate and dexterous application of memory retrieval strategies, effective learning strategies or methods are required to guide children to encode arithmetic facts in long-term memory (Adesope et al., 2017; Agarwal et al., 2021; Ophuis-Cox et al., 2023). This study exactly employs a schema-based method to assist them in achieving this and investigates its specific effects on rectifying persistent errors. The following sections will further elaborate on the theories related to a schema-based method.

### 2.3 Mental arithmetic and schema theory

In his research, Bartlett found that participants tend to use their familiar schemas to understand new information, so he defined schema as “an active organization of past reactions or experiences of the individuals” (Bartlett and Bartlett, 1995). Piaget defined schema as “a structure or organization of actions, which, through repetition in similar or analogous situations, leads to the transfer or generalization of these actions” (Piaget, 2002), and demonstrated the critical role of schema in thinking and creative processes. Brown and Yule (1983) argued that schemata are well-organized background knowledge that guides the prediction of discourse content. In the 1970s, psychologist Rumelhart developed the concept of schemata in his schema theory which categorized schemata into three types: linguistic schemata, rhetorical schemata, and content schemata (Carrell and Eisterhold, 1983). Nowadays, the schema has become a core concept in cognitive psychology, widely applied in memory research to help understand advanced cognitive processes such as thinking.

The automation of schema and rule automation do not require additional resources and can compensate for the limited capacity of an individual’s working memory, reducing cognitive load and improving transferability and transfer stability. In cognitive development theory, mental schema play an important role in cognitive processes, allowing for the construction of past experiences in a simplified manner, and allowing for spontaneous filtering, sorting, and organizing of external stimuli in new contexts to form organized modular knowledge structures. Through the recall of prior knowledge and experiences, individuals can gain symbolic meanings and emotional experiences of objective objects (Simonton, 2000). Thus, strengthening the construction of schema can help learners establish a sound cognitive structure. As can be extrapolated from the above demonstration, schema-based memory is highly efficient. Therefore, this study adopts the schema-based mental arithmetic method to correct persistent arithmetic errors in the participants’ memories, and subsequently examines whether the phenomenon of persistent errors is improved.

**Figure 1:**
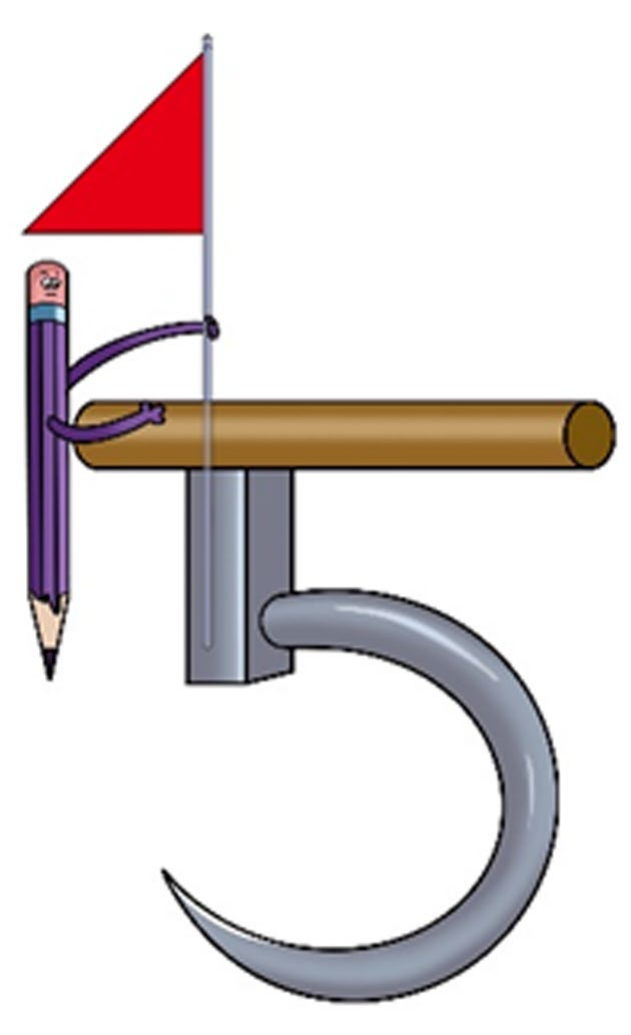
The image for equation 4 + 1 = 5.

According to the schema theory, schema is a high-level knowledge structure formed based on long-term memory, which represents an abstraction of things in life that a learner has experienced. Schema can bind multiple elements simultaneously, with overlapping and associative relationships being built between the elements (Gilboa and Marlatte, 2017). Therefore, based on the characteristics of numbers, students’ cognitive features, and memory rules, the numbers in the arithmetic equation are encoded as real-life elements, and the links among the elements are also established to simulate the arithmetic relationships among the numbers. As shown in Figure 1, the red flag represents the number “4”, the pencil represents the number “1”, and the weighing hook represents the number “5”. The equation represented by this image is 4 + 1 = 5.

The schema-based mental arithmetic method not only possesses the superiority of schema but also visualizes arithmetic facts in an image-oriented manner, enabling students to connect mathematical expressions with real-life objects, thereby enhancing their memory retention. In the context of visualization research, in the late 1960s to early 1970s, Canadian psychologist Paivio (2013) proposed the Dual Coding Theory. He posited that the human cognitive system typically comprises two coding systems—verbal coding and non-verbal coding. The two systems possess different forms and representational units for information perception. Experimental evidence indicates that a non-verbal coding system exhibits outstanding performance in the extraction and free recall of information. For instance, reading combining text and images show markedly higher efficiency compared to reading pure text. Different encoding of explicit knowledge plays distinct roles in the dissemination of information among individuals.

The essence of visual representation of knowledge lies in the explicit visual coding of knowledge products. The visual and the auditory coding of explicit knowledge products provide complements for each other, and simultaneously fundamental impetus together for visualized thinking. Eppler and Burkhard (2008) defined “knowledge visualization” as: “In general, the field of knowledge visualization research focuses on the application of visual representation in improving knowledge creation and transmission between two or among more individuals.” Therefore, knowledge visualization can be considered as the application of visual representation to present knowledge in the form of charts, assisting in instructional design, and promoting the reflections of both teachers and students in teaching activities. Whether in traditional teaching with language as the primary medium or in visualization teaching aided by multimedia, the ultimate goal is to facilitate learners’ knowledge acquisition and skill enhancement.

Thus, based on the schema theory, this paper just adopts the visualized strategy that uses images to present arithmetic facts as shown in Figure 1. Meanwhile, the images can stimulate neural activity through association, resulting in a significant enhancement in memory. This is because images enable learners to establish meaningful connections between visual and verbal information (Ahmadi and Zarei, 2021). Numerous studies indicate that transforming materials required to be memorized into visually intuitive images often results in better memory and recognition of images compared to mere text, highlighting the advantage of image memory (Ritchey, 1980). Image memory enables learners to engage in more cognitive activities, and deepens the information processing, rendering memories more stable and robust (Craik and Lockhart, 1972; Kobayashi, 1986).

## 3 Methods

### 3.1 Participants

The present study focused on elementary school students in grades 4, 5, and 6. The reason for the selection of these participants is to ensure that all the participants will use more memory retrieval strategies when performing calculations. Specifically, we selected 120 students from primary schools in rural areas of Tianmen City (in Hubei Province, China). These students were randomly assigned to either the experimental group or the control group, with each group comprising 60 students. The experimental group consisted of 25 boys and 35 girls, and the control group had 26 boys and 34 girls. The average ages of the control group and the experimental group are 10.06 and 10.25, with standard deviations of 0.97 and 1.34, respectively, indicating similarity in age between the two groups. The gender ratios for the two groups are 0.71:1 and 0.76:1, respectively, demonstrating similarity in gender distribution, as well. Additionally, all the participants have a similar level of educational attainment. In summary, the participants in the control group and experimental group are comparable in terms of demographic characteristics.

### 3.2 Instruments

#### 3.2.1 Design of a test paper for single-digit addition

This experiment used an addition scale (refer to Appendix 1). The scale consisted of six sets of single-digit addition test questions, with the same questions in each set but in a different sequence of augend and addend, which were used to count the number of errors made by students on the same question and to detect the occurrence of persistent arithmetic errors. The test questions on this scale were used for both pretest and post-test in the experiment. But to avoid bias, the order of the six sets of test questions for the pretest and post-test were randomly assigned.

#### 3.2.2 Creation of the schema-based teaching aids

Based on the cognitive load theory, knowledge exists in the form of cognitive schema in human’s long-term memory. The purpose of learning is to intensify the training, automate cognitive activities, and reduce the cognitive load of an individual. The development of mental arithmetic schema teaching aids is to visualize the most basic combinations/blocks of arithmetic in mental arithmetic. The first step is to encode the numbers into common physical images through images or homophonic ways. For instance, the number “7” is shaped like a “sickle,” the number “9” is shaped like a “balloon,” and the pinyin “SHÍ LIÙ” for the number “16” can be encoded as a homophone of “SHÍ LIU” (pomegranate). The second step is to integrate the coded images of the three numbers in the mental arithmetic formula into a single picture through a brief story. It should be noted that the images of attend and augend should be significantly smaller than the image of the “sum.” For example, the formula 7 + 9 = 16 has three image elements (i.e., “sickle,” “balloon,” and “pomegranate,” respectively), and the image of the pomegranate should be obviously larger than the other two. Next, combined with a story that “a sickle from the giant pomegranate dug out a balloon “, the final graphic aid has been developed with these images.

**Figure 2:**
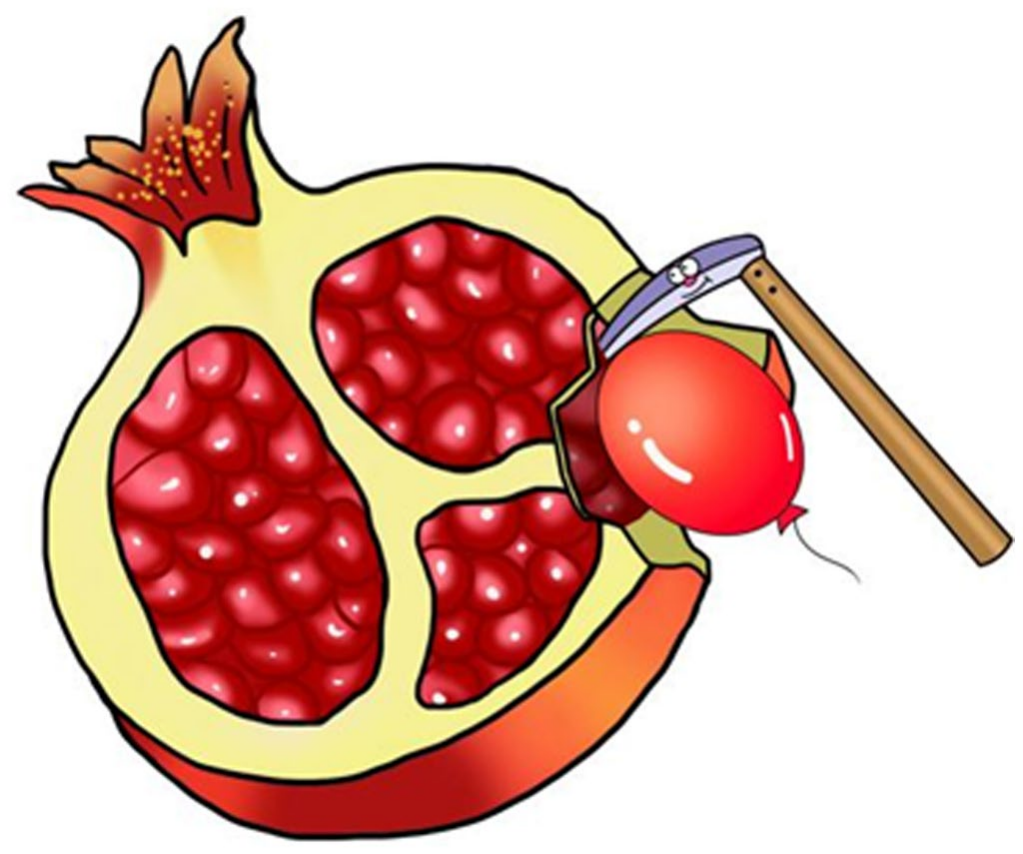
The image for equation 7 + 9 = 16.

We have developed a total of 45 schemata (see Appendix 1) ranging from 1 + 1 = 2 to 9 + 9 = 18, arithmetic pictorials that contain all single-digit addition. In addition, because each graphic aid distinguishes between “addition” and “sum” by the size of the image, each schema actually represents four equations, e.g., Figure 2 represents 7 + 9 = 16, 9 + 7 = 16, 16–7 = 9, and 16–9 = 7. Compared to traditional mental arithmetic, the advantage of schema is manifested not only in reducing cognitive load and rendering learning process more interesting, but also in that it can integrate elements such as kinesthetic sense, five senses, images, interest, and uniqueness, etc., so as to deepen the connection to the working memory and transform the working memory to the long-term memory, thus enhancing the retention of the memory. What’s more, such schema-based memorization is error-free and helpful in correcting persistent errors.

The employment of these schema-based aids for mental arithmetic teaching is relatively simple. First of all, let the students be familiar with and memorize the number codes 1–18. Secondly, present the mental arithmetic schemata one by one to the students and encourage the students to create their own stories or scenarios according to the schemata (9 for each group) to mobilize the initiative of the students to deepen their working memory connection. Thirdly, ask the students to revert the schema to arithmetic equations according to the size and category of the elements; Finally, through calculation training, improve the students’ proficiency in the inter-converting between schemata and arithmetic equations.

### 3.3 Experimental procedures

Before the experiment, a pretest was conducted on the experimental and control groups to identify the students’ arithmetic errors. Research has shown that under the instruction to emphasize speed, students are more likely to use memory retrieval strategies (Ashcraft, 1982). Therefore, during the test, the instruction “at your fastest speed” was given, ensuring that most students chose memory retrieval strategies while doing calculations. Then, preliminary statistics were conducted on the results of the pretest, and students who exhibited persistent errors were selected for further experiments. In the experiment, a controlled trial was conducted. Graphic teaching aids were adopted in the experimental group used for simple addition teaching, where the teacher described and explained each graphic teaching aid and asked the students to repeat them for 20 min every day for a total training period of 15 days. The control group did not receive any training. Tests on the two groups were conducted in different classrooms, and teachers also took supervisory roles to prevent communication between the two groups of students. This effectively prevented the two groups from discovering their differences and achieved the placebo effect. After the experiment, a post-test was conducted on both groups, during which the instruction “at your fastest speed” was given again. The experimental group used graphic teaching aids. They used the schema-based method to rectify persistent errors in memory. By comparing the experimental group with the control group, if the former performs better, it proves that students’ persistent errors in mental arithmetic are related to the interference of incorrect answers in memory retrieval.

### 3.4 Data collection and analysis

In this study, persistent errors were counted separately in the pretest and post-test for both the experimental and control groups. A fixed incorrect answer that emerged two or more times for the same question was considered as a persistent error. For example, if a student answered 3 + 5 = 7 three times, 8 + 1 = 10 two times, and 2 + 5 = 8 two times, he would be recorded as having made three persistent errors. Statistical analysis was conducted with the help of SPSS software on four variables:

1. group: experimental and control groups;
2. the number of persistent errors made in the pretest by each student;
3. the number of persistent errors made in the post-test by each student;
4. the reduction in the number of persistent errors from pretest to post-test.

First of all, a significance test was conducted on the pretest results of both groups to ensure that the initial arithmetic levels of students were comparable. Likewise, a significance test was also needed in terms of the post-test results of both groups. Finally, one more significance test was made on the reduction in the number of persistent errors between the experimental and control groups to further examine the experimental effect.

## 4 Experimental results

Both the experimental group and the control group consist of 60 individuals. A preliminary analysis was conducted on the number of students whose pretest results showed persistent errors. It turned out to be that 16 participants in the experimental group (26.67%) and 17 participants in the control group (28.33%) exhibited persistent errors during the pretest. The similar percentage of participants facing persistent errors in both groups in the pretest indicated that there was indeed a phenomenon of persistent arithmetic errors among students and that the initial arithmetic levels of the two groups were comparable. Next, the students with persistent errors were picked out to form the experimental and control groups which were comprised of 16 and 17 students, respectively. The assignment would remain unchanged for subsequent experiments.

### 4.1 Significance test

#### 4.1.1 Pretest evaluation

To begin with, whether the data for the number of errors in the pretest was normally distributed was checked. Since the sample size was *N* = 33 < 5,000, we used the Shapiro–Wilk test at first and yielded the *W*-value (0.60), *p* < 0.001, which indicated that the data did not follow a normal distribution. Therefore, we turned to using the Mann–Whitney *U*-test for independent samples.

As shown in Table 1, both the median values for the pretest frequencies in the control group and experimental group are 1.0. The Median Absolute Deviation (MAD) of 0 indicates no difference between the two groups at the median level. Their respective mean values are 1.0 and 1.31, indicating a slight difference between the two groups at the mean level. The standard deviations are 0.61 and 0.60 for the control group and experimental group, suggesting a minimal disparity in the dispersion of data. The results of the U-test showed a *p*-value of 0.80 and a u-value of 141.5, indicating non-significance. The Cohen’s *d* value for the difference is 0.07. When Cohen’s *d* value is less than 0.2, it denotes a small effect; between 0.2 and 0.5, it signifies a medium effect; while around 0.8 or larger, a large effect is considered (Cohen, 1988). Therefore, Cohen’s *d* of 0.07 suggested a small effect size, indicating a very small difference. Consequently, there was no significant difference between the control and experimental groups in pretest frequencies, which showed that there was no substantial disparity in the initial arithmetic proficiency between the students in the two groups, ensuring that the experimental results were not unduly influenced by significant differences in students’ arithmetic proficiency.

**Table 1:** Analysis results table for pretest and post-test assessments.

Variable name	Variable value	Sample size	Mean	Standard deviation	*u*	*p*	MAD	Cohen’s *d*
The number of errors in the pretest	Control group	17	1	0.61	141.5	0.80	0	0.07
	Experimental group	16	1.31	0.60				
The number of errors in the post-test	Control group	17	1	0.66	231.5	<0.001	1	1.59
	Experimental group	16	0.31	0.48				

Significance level is set at 0.05, and a *p*-value less than 0.05 is considered statistically significant.

#### 4.1.2 Post-test evaluation

In the first place, the normality of the data was tested to determine whether it was normally distributed. Since the sample size for the number of errors was *N* = 33 < 5,000, the Shapiro–Wilk test was first adopted. The significance level was found to be *p* < 0.001, and the *W*-value was 0.79, indicating a significant departure from normality. Therefore, the Mann–Whitney *U*-test should be employed.

As shown in Table 1, the median values for post-test frequencies in the control group and experimental group are 1.0 and 0.0, respectively. The Median Absolute Deviation of 1 suggests a certain difference between the two groups at the median level. Their respective mean values are 1.0 and 0.31, indicating a significant difference between the two groups at the mean level. Meanwhile, the results from the experimental group exhibited mean values of 1.31 and 0.31 in the pretest and post-test, respectively, suggesting a reduction in error frequencies, and thus a favorable outcome. The standard deviations are 0.66 and 0.48 for the control group and experimental group, signifying a certain level of disparity in the dispersion of data between the two groups. The data distribution in the experimental group is relatively concentrated. The *U*-test results yielded a *p*-value of less than 0.001, and the u-value was 231.5, demonstrating statistical significance. The effect size, measured by Cohen’s *d*, was 1.59, denoting a very large magnitude of difference. Therefore, there was a significant difference between the control and experimental groups in post-test frequencies. It can be easily observed that the graphic aids have effectively corrected the persistent errors in the memory of experimental group students. Furthermore, as most students rely on memory retrieval when doing mental arithmetic, it provided proof from the reverse side for the former hypothesis that the occurrence of persistent errors in the mental calculating process is associated with interference from incorrect answers in memory retrieval.

#### 4.1.3 Test on reduction of error count

Primarily, the data was checked whether it followed a normal distribution with the Shapiro–Wilk test since the sample size of the error reduction count was *N* = 33 < 5,000. The *W*-value was 0.82, *p* < 0.001, indicating a significant departure from normality and negating the null hypothesis. Likewise, we conducted a Mann–Whitney *U*-test then.

As shown in Table 2, the median reduction in the number of errors for the control group and the experimental group are 0.0 and 1.0, The Median Absolute Deviation of 1 suggests a marked difference between the two groups at the median level. Their means are 0 and 1, with the experimental group exhibiting more reduction in the number of errors compared to the control group. This indicated there was a significant decrease in error rates for the experimental group, suggesting a positive experimental effect. Their standard deviations were 0.49 and 0.63, indicating a certain level of disparity in the variability between the two groups, with the data distribution in the control group being relatively concentrated. The test result was *p* < 0.001, and the *u*-value is 43.5, indicating statistical significance. The effect size, Cohen’s *d* value, was 1.572, which suggests a very large difference between the control and experimental groups in terms of decreased errors. These results demonstrate jointly that the experimental group’s persistent errors were significantly reduced with the help of the schema-based teaching aids whereas the control group’s persistent errors remained largely unchanged. This further confirms the initial hypothesis.

**Table 2:** Mann–Whitney *U*-test for reduction in the number of persistent errors for the control and experimental groups.

Variable name	Variable value	Sample size	Mean	Standard deviation	*u*	*p*	MAD	Cohen’s *d*
The reduction in the number of errors	Control group	17	0	0.49	43.5	<0.001	1	1.57
	Experimental group	16	1	0.63				

Significance level is set at 0.05, and a *p*-value less than 0.05 is considered statistically significant.

## 5 Discussion and conclusion

### 5.1 Discussion

This study aimed to investigate the effect of the schema-based method in correcting students’ persistent errors in mental arithmetic practice. Through results analysis of a controlled experiment, the results revealed that correcting students’ persistent errors in mental arithmetic through the schema-based method is effective. Despite the initial similarity between the experimental and control groups in pretests, significant disparity was found in post-tests. And the reduction degree in the number of errors also showed significant differences. This illustrates a significant decrease in persistent error rates in the experimental group during the post-test, unraveling a positive experimental effect compared to the control group. It points to the fact that the experimental group effectively corrected persistent arithmetic errors in memory through the schema-based method.

Although Myers (1924) had ever studied similar error phenomena, the cause of the phenomenon was ignored. This study, through a controlled experiment, testified the hypothesis in a reverse way and investigated the persistent errors which were not ever mentioned in previous studies on arithmetic error types (Engelhardt, 1977; Radatz, 1979; Herholdt and Sapire, 2014; Sarwadi and Shahrill, 2014; Muthukrishnan et al., 2019). When a student makes an arithmetic error only once, it may be a random one. However, when he gives the same incorrect answer multiple times for the same arithmetic problem, it is unlikely to be random. Therefore, the author hypothesized that such persistent errors in mental calculation are caused by interference from incorrect answers in memory retrieval. This hypothesis is based on the fact that students are apt to retrieve answers from memory (memory retrieval) while doing mental calculations. There have been many studies on memory retrieval models for mental calculation and arithmetic error types, but none has linked memory retrieval models to persistent errors in mental calculation before this study. In this research, by rectifying the incorrect answers in memory retrieval, the negative interference can be removed and the persistent arithmetic errors can be rectified. Meanwhile, the accuracy and efficiency of mental arithmetic can be improved, which would provide significant help for teaching and learning activities.

The schema-based mental arithmetic method is implemented through non-verbal encoding by visualizing arithmetic facts and connecting mathematical concepts with real-life objects for students. The use of schema can be viewed as a form of non-verbal encoding, aligning with the relevant theory proposed by Paivio (2013). In the learning process, through visual representation, students can gain a deeper understanding and memorization of arithmetic facts, as the images can provide meaningful associations and contexts. The concept of knowledge visualization further supports the schema-based mental arithmetic method. In this method, presenting arithmetic facts with images is exactly an application of knowledge visualization. It can not only aid teachers in the designing of more effective instruction but also encourage students to actively make reflections during educational activities. By combining graphic design and cognitive science, the schema-based mental arithmetic method endows the students with a more engaging and efficient learning way. Moreover, the research results mentioned in this paper support the advantages of visual mnemonics. Transforming material that needs to be memorized into visually intuitive images helps deepen information processing and enhance memory stability. The emphasis on visual memory in cognitive activities diverges from traditional text-centered teaching. To be specific, the positive role of visual memory in learners’ cognitive activities is highlighted, aligning with the schema theory that focuses on perception and cognition in learning.

As for why visual representation of numbers through images can be feasible and effective, the underlying cognitive mechanisms warrant more consideration. This study hypothesizes a potential association with the brain’s associative functions. O'Malley and Chamot (1990) proposed associative memory strategies, referring to linking new information with acquired knowledge or establishing connections between individuals and information to enhance understanding and memorization of learning materials, similar to the schema-based method employed in this study, in which numbers are linked to daily experiences and objects by resorting to images that closely resemble real-life objects to represent every single number and combining these images to form mental representations of arithmetic facts. Consequently, students can automatically convert these images into arithmetic facts through association and store them in long-term memory. When solving similar arithmetic problems next time, students can swiftly associate these mental representations of arithmetic facts to retrieve answers.

### 5.2 Unique contribution and future outlook

Many scholars have engaged in in-depth discussions on the best teaching methods for mathematics, giving rise to a variety of instructional strategies (Gulson, 2021). The unique contribution of this study is evident in its profound impact on future educational strategies. Teachers can actively apply schema-based teaching tools, and integrate them into mental arithmetic instruction, to help students correct persistent errors and enhance their mental arithmetic abilities. This study provides valuable guidance, practical and theoretical, to arithmetic education. By further exploring these findings, a more comprehensive understanding of the learning process can be gained, which helps lay a solid foundation for the advent of more innovative teaching methods and strategies, thus driving continuous progress in the field of arithmetic education. In the future, emphasis should be placed on teacher training to enhance their proficiency in schema-based teaching. As teachers play a guiding role in the implementation of schema-based arithmetic teaching, their expertise and teaching skills are essential for the success of schema-based instruction. Additionally, further research should delve into the impact of schema-based teaching on students’ learning interests, motivation, and attitudes, as well as how to stimulate students’ initiative and self-directed learning through schema-based instruction. This will contribute to optimizing relevant pedagogy, and improving student’s learning experiences and performance.

This study simultaneously fills the gaps in previous research. Scholars have explored the impact of schema-based instruction on mathematical problem-solving abilities (Fuchs et al., 2004). Xin’s (2008) research aimed to explore the effects of schema-based teaching strategies on the arithmetic applications of algebraic concepts among primary school students who encounter learning obstacles or issues (LP). Mudrikah and Hakim (2016) implemented Problem-Based Learning (PBL) combined with Action, Process, Object, Schema (APOS) theory for 26 prospective mathematics teachers. Gembong and Suwarsono (2018) study aimed to investigate the schemata established by elementary school students while solving fraction addition problems. In Gulson’s study Gulson (2021), teachers can embed abstract concepts like equation solving within students’ existing knowledge structures using schema theory. In summary, schema theory has garnered extensive attention in the field of mathematics education. However, there has been relatively less specific application of schema theory in correcting continuous errors in mental arithmetic within these studies. Our research fills this knowledge gap, underscoring the potential value of schema theory in rectifying mental arithmetic. Furthermore, our study’s outcomes not only confirm the effectiveness of schema-based methods in mental arithmetic but also advance the development of schema theory. Through experimental results, we may offer new insights into how schema influences the cognitive processes and learning outcomes in mental arithmetic. This paves an interesting path for future research, delving deeper into the specific mechanisms of schema-based methods in mental arithmetic and potentially triggering more profound theoretical discussions on mental arithmetic instructional approaches.

Despite valuable findings yielded in this study, there are still various shortcomings in its research design and results analysis. Firstly, in the experiment, the relatively small number of participants who are primarily concentrated in rural schools in fixed regions limits the external validity of the results. Future research can enhance external validity by increasing the number of participants and diversifying the distribution of source regions. Additionally, extending the testing period and conducting long-term observations can more comprehensively validate the long-standing reliability of experimental results. Moreover, the experiment still embraces uncontrolled factors, such as the intentional behaviors of students, which may have influenced the results more or less. To better understand the results, future research can employ more strict and detailed control measures to eliminate potential confounding factors. While this study confirms the effect of the schema theory in addressing persistent errors in mental arithmetic, the applicability to other error types remains to be validated.

This study intends to further develop schema-based teaching tools in different subjects under existing conditions and explore the application effects and implementation strategies of schema-based teaching in various subjects and grade levels. Since the characteristics and requirements of learning differ across subjects and grades, customized design and optimization of schema-based teaching models become necessary. In the development of teaching aids, with the rapid advancement of information technology, visualization techniques and tools have gained widespread application (Yuancheng, 2018). Future research directions may include how to effectively combine visualization techniques with schema-based teaching to create more diverse and personalized teaching resources and tools. In summary, valuable insights can be provided for future research and educational practices by reviewing the limitations of this study, and guidance for further advancement in this field is also available.

## Data availability statement

The original contributions presented in the study are included in the article/supplementary material, further inquiries can be directed to the corresponding author.

## Ethics statement

The studies involving humans were approved by cognitive psychology of Brain Science and Learning Science Committee of Hubei Teachers Education Association. The studies were conducted in accordance with the local legislation and institutional requirements. Written informed consent for participation in this study was provided by the participants’ legal guardians/next of kin. The animal study was approved by cognitive psychology of Brain Science and Learning Science Committee of Hubei Teachers Education Association. The study was conducted in accordance with the local legislation and institutional requirements.

## Author contributions

SC: Data curation, Writing – original draft. DL: Methodology, Writing – review & editing. HY: Resources, Writing – review & editing. YM: Writing – original draft.
