# Supplementary figures and images for: The effect of a Schema-based method on correcting persistent errors in mental arithmetic: an experimental study
Source: Front Psychol. 2024 May 20;15:1276914. doi: 10.3389/fpsyg.2024.1276914 (PMC11146372; doi:10.3389/fpsyg.2024.1276914)

# Appendix 1

| 姓名 (name)   |             | 年级 (grade)  |             | 日期 (date)   |             |
|-------------|-------------|-------------|-------------|-------------|-------------|
| 6+3=        | 5+4=        | 6+4=        | 6+1=        | 6+2=        | 4+2=        |
| 4+2=        | 6+2=        | 4+4=        | 6+3=        | 4+3=        | 2+2=        |
| 3+3=        | 4+3=        | 7+2=        | 5+1=        | 3+3=        | 4+1=        |
| 1+7=        | 8+1=        | 3+3=        | 4+2=        | 6+1=        | 1+2=        |
| 7+2=        | 4+2=        | 2+5=        | 6+2=        | 6+3=        | 8+2=        |
| 6+1=        | 2+2=        | 1+9=        | 4+3=        | 5+1=        | 3+3=        |
| 5+4=        | 3+1=        | 3+2=        | 3+3=        | 4+2=        | 6+1=        |
| 3+1=        | 6+4=        | 6+1=        | 2+5=        | 4+4=        | 6+3=        |
| 1+9=        | 4+4=        | 6+3=        | 1+9=        | 7+2=        | 2+5=        |
| 8+1=        | 4+1=        | 5+1=        | 3+2=        | 2+5=        | 1+9=        |
| 6+2=        | 1+2=        | 1+7=        | 6+4=        | 1+9=        | 3+2=        |
| 4+3=        | 8+2=        | 8+1=        | 4+4=        | 3+2=        | 5+1=        |
| 3+5=        | 5+1=        | 4+2=        | 7+2=        | 6+4=        | 1+7=        |
| 4+1=        | 1+7=        | 6+2=        | 4+1=        | 3+1=        | 7+2=        |
| 1+2=        | 7+2=        | 4+3=        | 1+2=        | 3+5=        | 3+1=        |
| 8+2=        | 3+3=        | 5+4=        | 8+2=        | 1+7=        | 6+4=        |
| 5+1=        | 6+1=        | 4+1=        | 2+2=        | 4+1=        | 4+4=        |
| 3+2=        | 6+3=        | 1+2=        | 3+1=        | 1+2=        | 4+3=        |
| 6+4=        | 2+5=        | 8+2=        | 3+5=        | 8+2=        | 8+1=        |
| 2+5=        | 1+9=        | 2+2=        | 1+7=        | 2+2=        | 5+4=        |
| 4+4=        | 3+2=        | 3+1=        | 8+1=        | 8+1=        | 6+2=        |
| 2+2=        | 3+5=        | 3+5=        | 5+4=        | 5+4=        | 3+5=        |
| 时间 (time) : |

## Appendix 2

|                                                                                     |                                                                                     |                                                                                      |                                                                                       |
|-------------------------------------------------------------------------------------|-------------------------------------------------------------------------------------|--------------------------------------------------------------------------------------|---------------------------------------------------------------------------------------|
| 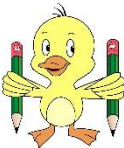   | 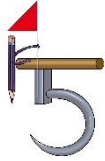   | 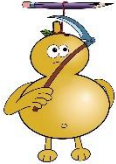    | 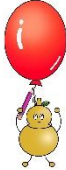   |
| $1+1=2$                                                                             | $1+4=5$                                                                             | $7+1=8$                                                                              | $8+1=9$                                                                               |
| 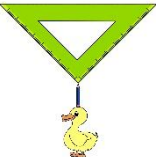   | 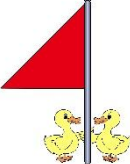   | 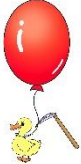    | 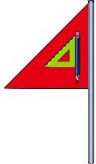   |
| $1+2=3$                                                                             | $2+2=4$                                                                             | $2+7=9$                                                                              | $1+3=4$                                                                               |
| 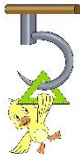   | 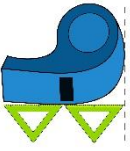   | 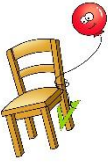    | 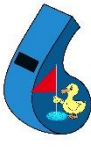   |
| $3+2=5$                                                                             | $3+3=6$                                                                             | $3+9=12$                                                                             | $4+2=6$                                                                               |
| 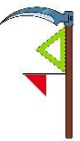 | 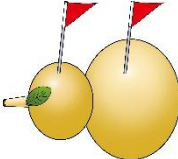 | 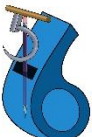  | 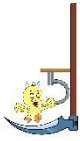 |
| $4+3=7$                                                                             | $4+4=8$                                                                             | $5+1=6$                                                                              | $5+2=7$                                                                               |
| 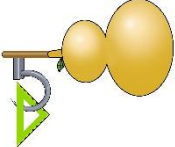 | 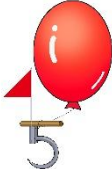 | 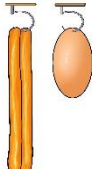  | 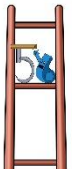 |
| $5+3=8$                                                                             | $5+4=9$                                                                             | $5+5=10$                                                                             | $5+6=11$                                                                              |
| 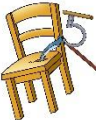 | 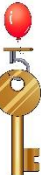 | 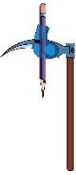  | 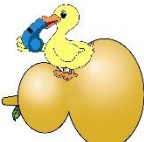 |
| $5+7=12$                                                                            | $5+9=14$                                                                            | $6+1=7$                                                                              | $6+2=8$                                                                               |
| 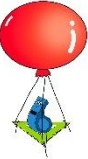 | 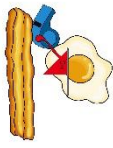 | 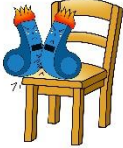 | 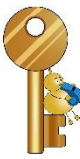 |
| $6+3=9$                                                                             | $6+4=10$                                                                            | $6+6=12$                                                                             | $6+8=14$                                                                              |

|                                                                                     |                                                                                    |                                                                                    |                                                                                      |
|-------------------------------------------------------------------------------------|------------------------------------------------------------------------------------|------------------------------------------------------------------------------------|--------------------------------------------------------------------------------------|
| 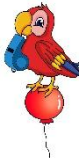   | 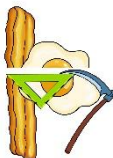  | 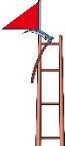  | 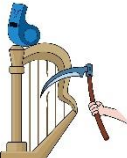  |
| $6+9=15$                                                                            | $7+3=10$                                                                           | $7+4=11$                                                                           | $7+6=13$                                                                             |
| 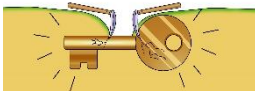   | 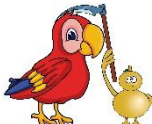  | 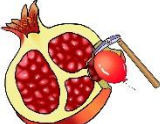 | 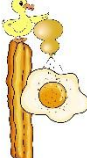  |
| $7+7=14$                                                                            | $7+8=15$                                                                           | $7+9=16$                                                                           | $8+2=10$                                                                             |
| 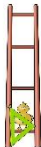   | 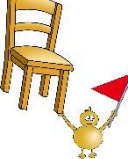  | 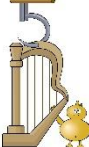  | 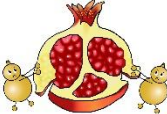  |
| $8+3=11$                                                                            | $8+4=12$                                                                           | $8+5=13$                                                                           | $8+8=16$                                                                             |
| 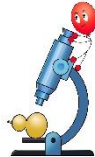  | 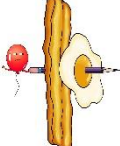 | 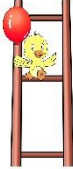 | 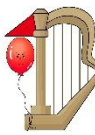 |
| $8+9=17$                                                                            | $9+1=10$                                                                           | $9+2=11$                                                                           | $9+4=13$                                                                             |
| 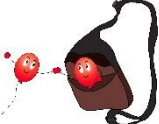 |                                                                                    |                                                                                    |                                                                                      |
| $9+9=18$                                                                            |                                                                                    |                                                                                    |                                                                                      |

Supplement: Supplementary file 1 [file Presentation_1.pdf]
